# The Gynecic Use of Hydrochlorate of Cocaine

**Published:** 1884-12

**Authors:** 


### The Gynecic Use of Hydrochlorate of Cocaine.

This new local anæsthetic, which has met with considerable success in the hands of ophthalmologists and laryngologists, has made its way into the field of gynecology, and the results of the few experiments already made with it are such as to promise considerable usefulness in operations upon the genital tract of woman, as well as in many other minor surgical procedures.

Dr. William M. Polk, of New York, on the 29th of October, 1884 (*Medical Record*, Nov. 1, 1884), closed rents of the cervix uteri in two cases, after painting over the cervix a four per cent. solution, with a camel's-hair brush. The application was also carried within the cervical canal and over the adjacent vaginal wall; three applications of the drug were made, at intervals of two or three minutes, and within three minutes of the last application the operations were begun. The first was an elaborate operation, requiring forty minutes to make, and the patient made no complaint of pain until the last ten minutes, when her discomfort was rather a sense of soreness than acute pain.

In the second case, there was considerable normal sensitiveness of the sexual organs, and less self-control on the part of patient. No pain, however, was felt until the lapse of twenty minutes, when it became so acute as to require another application (the fourth) which was made directly to the cut surfaces, after freeing them from blood.

The testimony of this patient is valuable, as she had, three years previously, undergone the same operation with ether, and when asked which method she preferred, she promptly answered, the last.

Another effect which Dr. Polk observed was, that the first appearance of blood upon the cut surfaces was retarded, which would tend to show that the new local anæsthetic also possessed some hæmostatic properties.

The recorded results reached with this new drug are yet too few for us to make definite deductions therefrom, but as experiments with it aggregate we shall lay the data before our readers from time to time. It is already being tested in reference to its effects upon the urethra, rectum, and other mucous membranes, in addition to its uses noted above, and the results thus far obtained are such as to justify a belief that it will prove of value in other directions as yet untried. We invite reports from any of our readers who may test the merits of this remarkable drug.

